# TET1-mediated DNA demethylation and transcription activation of MRPS17 induces lung adenocarcinoma through PI3K-AKT-mTOR pathway

**DOI:** 10.1007/s10735-026-10862-8

**Published:** 2026-08-29

**Authors:** Nan Zhou, Lei Song, Yang Wu, Ying Wei, Wenwen Zhang, Bingbing Wang, Yanbin Zhao, Yan Wang

**Affiliations:** 1 Department of Oncology, The First Affiliated Hospital of Harbin Medical University, 23 Youzheng Street, Nangang District, Heilongjiang 150001 Harbin, China; 2 Department of Respiratory Oncology, Harbin Medical University Cancer Hospital, 150 Haping Road, Nangang District, Harbin, 150040 Heilongjiang Province China; 3 Breast Surgery, Harbin Medical University Cancer Hospital, Harbin, 150040 Heilongjiang Province China; 4 Department of cardiology, The First Affiliated Hospital of Harbin Medical University, Harbin, 150001 Heilongjiang Province China; 5 Surgical Department of Gynecologic Oncology, Harbin Medical University Cancer Hospital, Harbin, 150040 Heilongjiang Province China

**Keywords:** LUAD, MRPS17, PI3K-AKT-mTOR

## Abstract

Lung adenocarcinoma (LUAD), characterized by its complex molecular heterogeneity and resistance to both conventional and targeted therapies, poses significant therapeutic challenges. Therefore, the identification of novel molecular targets is crucial for enhancing therapeutic strategies and improving patient outcomes. This study investigates the role of mitochondrial ribosomal protein S17 (MRPS17) in the progression of LUAD, with a specific focus on its interaction with the PI3K-AKT-mTOR signaling pathway. We conducted functional assays to assess cell proliferation, migration, invasion, and apoptosis in MRPS17-manipulated LUAD cell lines. Further, we explored the epigenetic regulation by TET1 through methylation analysis and investigated the downstream effects on the PI3K-AKT-mTOR pathway using Western blotting and reporter assays. Analysis revealed that MRPS17 is upregulated in LUAD tissues and is associated with a poor prognosis. In cellular models, MRPS17 overexpression was shown to promote proliferation, migration, and invasion, whereas its knockdown induced apoptosis and diminished tumorigenic capabilities both in vitro and in vivo. Importantly, TET1 was identified as a crucial regulator of MRPS17, acting through the demethylation of its promoter to enhance MRPS17 expression and subsequently activate the PI3K-AKT-mTOR pathway. MRPS17 significantly contributes to LUAD progression by enhancing tumor aggressiveness through the PI3K-AKT-mTOR pathway. The TET1-mediated demethylation of MRPS17 introduces a novel epigenetic mechanism that could be leveraged for targeted therapeutic interventions. This study not only provides foundational insights into the molecular biology of LUAD but also highlights the potential of MRPS17 as a prognostic marker and therapeutic target.

**Supplementary Information:**

The online version contains supplementary material available at https://doi.org/10.1007/s10735-026-10862-8.

## Introduction

Lung cancer is the leading cause of death from cancer in humans, with 1.76 million related deaths each year (Sung et al. 2021). Lung adenocarcinoma (LUAD) is the most common of all types of lung cancer, accounting for 40% of cases (Naranjo et al. 2022). Early LUAD patients often have atypical symptoms, so they are not easy to diagnose, resulting in the majority of LUAD patients having metastasis when diagnosed (Román et al. 2018). At present, the main treatment strategy for LUAD is still surgical resection combined with chemotherapy, radiotherapy, immunotherapy and targeted therapy. However, only a small proportion of patients are clinically suitable for surgical treatment, the efficacy of traditional chemotherapy is limited, the side effects of radiotherapy are considerable, and the postoperative recurrence rate is high. Therefore, its treatment is still an important problem for medical researchers (Zhao et al. 2022). With the development of molecular biology, molecular targeted therapy for cancer has received increasing attention, and it is one of the important hot areas of cancer-related research (Liu et al. 2022, Chen et al. 2022). LUAD is a heterogeneous disease due to the different molecular mechanisms driving lung carcinogenesis, and the development of targeted molecular therapies has greatly improved patient response to treatment strategies (Yu et al. 2021, Yu et al. 2021, Imyanitov et al. 2021, Tan and Tan 2022, Passiglia et al. 2022). In recent years, targeted therapy for LUAD has made significant progress. The common therapeutic targets for LUAD include EGFR, ALK, BRAF, KRAS, NTRK, and ROS1, and the emerging targets include MET, RET, and HER2. The emergence of a variety of new targeted drugs has greatly improved the prognosis of LUAD patients, and LUAD patients are rapidly moving towards individualized and precise treatment. However, in the process of targeted therapy, patients usually develop drug resistance, and once drug resistance occurs, the therapeutic effect is greatly weakened. Moreover, a large proportion of patients cannot receive targeted therapy because gene mutations of known targets are not detected. Therefore, we still need to explore more new molecular targets to provide more options and programs for personalized and precise molecular targeted therapy for LUAD patients. In this study, we explored the role of mitochondrial ribosomal protein S17 (MRPS17) in the malignant progression of LUAD and elucidated the potential molecular mechanisms involved to provide new targets and programs for precise targeted therapy for LUAD.

In the realm of cancer biology, epigenetic mechanisms, particularly DNA methylation, have emerged as key regulators of gene expression (Ferro Dos Santos et al. 2024). Alterations in the DNA methylation landscape are characteristic of various cancers, affecting tumor suppressor genes and oncogenes (Oladipo et al. 2024). TET1, a member of the Ten-Eleven Translocation (TET) family of dioxygenases, plays a pivotal role in active DNA demethylation (Zhang et al. 2023). It converts 5-methylcytosine (5mC) to 5-hydroxymethylcytosine (5hmC), thereby modulating gene expression. Although studies have begun to unravel the contributions of TET1 in cancers, including its context-dependent roles as both a tumor suppressor and an oncogene, the specific epigenetic mechanisms by which TET1 modulates key oncogenic pathways in LUAD remain underexplored (Zacapala-Gómez et al. 2024, Pires et al. 2023).

Mitochondria play a vital role in the survival of eukaryotic cells. The process of protein synthesis within mitochondria relies on mitochondrial ribosomes. Mitochondrial ribosomal proteins are encoded by the mitochondrial ribosomal protein-coding gene family, which includes 30 genes encoding the small and 50 genes encoding the large subunit of the mitochondrial ribosomal. Each of these genes performs its own function and together maintains the complete function of MRP (Gopisetty and Thangarajan 2016). An increasing number of studies have shown that MRP is an apoptosis-inducing factor and plays a key role in the induction of apoptosis. Apoptosis is essential in tumour therapy. Three MRPs associated with apoptosis have been identified, including MRPS29, MRPL41, and MRPS30. Two recent large-scale studies have compared the mitochondrial genome mutation profiles of normal tissues from the same patient with more than 30 tumour types from more than 2000 human cancers by parallel DNA sequencing (Ju et al. 2014; Stewart et al. 2015), these studies identified many somatic substitutions with strong replicative strand bias, and C→T and A→G inversions on the mitochondrial heavy chain suggested that mitochondrial polymerase G errors were the main cause of mutations. These findings clearly demonstrate that selective pressures to preserve mitochondrial genome function are prevalent in human cancers. In recent years, studies have revealed the potential role of multiple MRPs in lung cancer. The genes encoding MRPs, which are closely related to the proliferation and metastasis of cancer cells, are mutated in lung cancer (Kim et al. 2017). MRPL13 is highly expressed in non-small cell lung cancer tissues and cell lines and promotes the proliferation of cancer cells; thus, MRPL13 can be used as an independent tumour marker and candidate therapeutic target (Jing et al. 2021). Another study showed that high MRPL15 expression was associated with poor prognosis in NSCLC patients and that this gene was inversely correlated with immune infiltration (Zeng et al. 2021). MRPL42 is highly expressed in early LUAD tissues and cell lines and promotes the proliferation, migration and invasion of cancer cells. High MRPL42 expression predicts poor prognosis in LUAD patients (Ham et al. 2019). Therefore, studies on the molecular mechanism of MRP will provide more opportunities for targeted therapy.

MRPS17 is a gene in the S17P protein family that encodes the 28 S ribosomal protein in the S17P protein family (Hanlon et al. 2004). MRPS17 is involved in mitochondrial translation and plays a crucial role in oxidative phosphorylation (Baughman et al. 2009). Deficiencies in MRPS17 result in respiratory impairments, and the localization of MRPS17-GFP fusion within the mitochondria underscores the crucial role played by MRPS17 in mitochondrial oxidative phosphorylation, a vital component of aerobic respiration (Hanlon et al. 2004). MRPS17 has been reported to be involved in the pathogenesis of myxosarcoma and phosphoserine transaminase deficiency, and processes associated with MRPS17 include biogenesis and maintenance of organelles and translation of viral RNA (Cheong et al. 2020). The study by ZHOU et al. investigated the mechanism of MRPS17 in gastric cancer and the correlation between the expression level of MRPS17 and the prognosis of gastric cancer patients. MRPS17 expression was significantly increased in gastric cancer tissues and cells and was significantly correlated with poor prognosis in patients with gastric cancer. Further analysis revealed that MPRS17 is associated with the PI3K/AKT pathway and cell adhesion molecules, whose functions are mediated by the collagen-containing extracellular matrix and receptor ligand/regulatory activity. In vitro experiments showed that knockdown of MRPS17 inhibited the proliferation, migration and invasion of GC cells. In addition, MRPS17 may promote the malignant progression of gastric cancer by targeting the PI3K/AKT pathway (Zhou et al. 2021). Konstantinos et al. showed that abnormal expression of many mammalian mitochondrial ribosomal proteins, including MRPS17, was closely associated with reduced survival in NSCLC patients, while MRPS17 expression showed the opposite trend in healthy older adults (Prokopidis et al. 2022). The clinical relevance, biological role, and underlying molecular mechanisms of MRPS17 in LUAD remain unclear. Studies on the function and mechanism of MRPS17 in LUAD will provide new therapeutic targets for LUAD patients, provide more opportunities and options for patients who cannot receive targeted therapy or who are resistant to targeted therapy, improve the therapeutic effect and prognosis of such patients, and promote the development of precise targeted therapy for LUAD. This article investigates how MRPS17 influences the malignant progression of LUAD and the mechanisms through which it regulates LUAD development.

This study seeks to bridge these critical gaps by examining the hypothesis that TET1 mediates the DNA demethylation and transcriptional activation of MRPS17, subsequently inducing LUAD progression through the PI3K-AKT-mTOR pathway. We aim to delineate the epigenetic regulation of MRPS17 by TET1 and explore the downstream effects of this regulation on the PI3K-AKT-mTOR signaling axis in LUAD. Understanding these interactions could provide novel insights into the epigenetic modulation of lung adenocarcinoma and uncover potential therapeutic targets within this pathway.

## Materials and methods

### Data collection

A pancancer analysis of MRPS17 expression was performed from MRPS17 mRNA expression data in 18,102 normal and tumor tissue samples from The Cancer Genome Atlas (TCGA) and Genotypic Tissue Expression (GTEx) databases for 33 cancer types. Furthermore, a pancancer analysis of MRPS17 protein expression was carried out across ten different tumor types using data derived from the Clinical Proteome Tumor Analysis (CPTAC) database.

### Patient data and clinical tissue samples

This study included a total of 132 LUAD patients who were first diagnosed and underwent surgery from January 2015 to January 2017 in Harbin Medical University Cancer Hospital. This study was approved by the Ethics Committee of Harbin Medical University Cancer Hospital. A total of 132 LUAD patients met the following inclusion and exclusion criteria: (Sung et al. 2021) had not received any cancer-related treatment, such as chemotherapy or radiotherapy; and (Naranjo et al. 2022) were first diagnosed with LUAD in our hospital. The exclusion criteria were patients who (Sung et al. 2021) were unwilling to join the study or (Naranjo et al. 2022) had received cancer-related treatment before surgery (Román et al. 2018). Patients with undiagnosed pathology. Among them, 59 patients were ≥ 60 years old, and seventy-three patients were < 60 years old. There were 50 males and 82 females. According to TNM staging, 50 patients had stage I disease, 20 patients had stage II disease, and 62 patients had stage III disease. Eighty-five patients had a tumour size ≥ 3 cm, 47 patients had a tumour size < 3 cm, 74 patients had positive lymph node metastasis, and 58 patients had no lymph node metastasis.

### Immunohistochemistry (IHC)

The sections were subjected to a deparaffinization process using xylene, followed by dehydration with ethanol. The sections were then boiled in sodium citrate buffer. Next, the sections were sealed and then incubated with MRPS17 antibody (Proteintech group, 18881-1-AP, 1:100). The immunohistochemical assays were analyzed by two experienced pathologists. Scoring was performed using the IRS scoring system on a scale of 2–9. The intensity of staining was categorized into 4 levels: 1 (no staining), 2 (weak staining), 3 (moderate staining), and 4 (strong staining). The percentage of positively stained cells was classified into five different levels: 1 (< 5%), 2 (6–25%), 3 (26–50%), 4 (51–75%), and 5 (> 75%). A median immunohistochemical score of 5 was used as the cut-off value, and a score ≥ 5 was defined as high MRPS17 expression, while a score < 5 was defined as low MRPS17 expression.

### Cell culture and cell transfection

Shanghai Zishi Biotechnology Co. Ltd. (Shanghai, China) provided all of the cell lines utilized in the investigation, and NSCLC cell lines (A549, H1993, H1299, H460, H1650) and human lung bronchial epithelial (HBE) cells were included. The medium was 1640 medium supplemented with 10% fetal bovine serum (Gibco, Shanghai, China) and 1% penicillin‒streptomycin (Corning, Shanghai, China), and the cells were placed in an incubator at 37 °C and 5% CO2.

Cells in the logarithmic growth phase were collected and evenly spread into six-well plates (each well containing 300 000 cells) and transfected when the cell growth density per well was 60–70%. Configuration of transfection reagent: First, 5 ml of transfectant was added to 200 µl of jet prime buffer and vortexed for 10 s. Then, 4 ml of jet prime was added, and the mixture was vortexed for 10 s and left at room temperature for 10 min. The transfection vectors used included MRPS17 overexpression vectors (named the OE group), empty vectors (named the CTRL group), MRPS17 siRNA#1 (named the KD1 group), MRPS17 siRNA#2 (named the KD2 group), and a negative control of MRPS17 siRNA (named the NC group). Two millilitres of 1640 medium without serum, penicillin or streptomycin was added to each well, and then the prepared transfection reagent was added, gently blown, mixed, and placed in an incubator at 37 °C and 5% CO2. After 6 h, the medium in each well was replaced with 2 ml of 1640 medium containing 10% FBS and 1% penicillin/streptomycin. After 48 h, the cells were collected, and siRNAs and overexpression vectors were obtained from the corresponding organizations (Jikai Gene, Shanghai, China) packaged in plasmids.

### RNA extraction and quantitative real-time PCR

RT‒qPCR was employed to assess the expression level of MRPS17 in NSCLC cell lines (A549, H1993, H1299, H460, H1650) and HBE cells. As per the operating criteria of TRIzol (Thermo Fisher), RNA was extracted. A reverse transcription kit was used to convert RNA samples into cDNA by reverse transcription. Finally, the expression of MRPS17 was detected in individual cells by RT‒qPCR. The primers used were GAPDH-F: ACAACTTTGGTATCGTGGAAGG; GAPDH-R: GCCATCACGCCACAGTTTC; MRPS17(F): ATGTCCGTAGTTCGCTCATCC; MRPS17-R: CCTGGTCACTCTCACTTTAGCA. The results were analyzed by the 2 − ΔΔCT algorithm.

### Western blotting

The cells were fully lysed to extract the proteins. Proteins with different molecular weights were separated by SDS‒PAGE, transferred to PVDF membranes and blocked with QuickBlock (Beyotime) for 15 min. Primary antibody incubation was carried out overnight at 4 °C. The primary antibodies included GAPDH (Boster), PI3K, AKT, mTor, P-PI3K, P-AKT, p-mTor (Abcam) and MRPS17 (Proteintech Group). On day 2, the secondary antibody (Boster) was incubated for one hour at ambient temperature. Imaging was performed in a Tanon 5200 imaging system using ECL developer.

### Transwell assay

For successful transfection of A549 and 1993 cells, 2 × 10^4^ cells were added to the compartment (200 µL), with the lower compartment containing 1640 medium and 15% taurine serum (600 µl). After 24–48 h, the cells were fixed with methanol and dyed with crystal violet. For the determination of cell invasiveness, the bottom of the chambers was lined with matrix gel and the cells were placed in an incubator at 37 °C for 1 h.

### Cell proliferation assay based on CCK-8 assay

Following transfection, A549 and H1993 cells in their logarithmic growth phase were harvested, and 100 µl of medium containing 5 × 10^3^ cells was dispensed into 96-well plates. Subsequently, the CCK-8 (Biosharp) reagent was added to the cells to be tested for 30 min at 0 h, 24 h, 48 h and 72 h. Finally, the optical density (OD) value of each well was measured at a wavelength of 450 nm. Higher absorbance values indicate enhanced cell activity.

### TUNEL assay

The TUNEL assay was performed to detect apoptosis in LUAD cells using the In Situ Cell Death Detection Kit, Fluorescein (Roche, Cat# 11684795910). Initially, cells were fixed with 4% paraformaldehyde and permeabilized with 0.1% Triton X-100 in 0.1% sodium citrate. Following permeabilization, cells were incubated with terminal deoxynucleotidyl transferase and fluorescein-dUTP, provided in the kit, to label DNA strand breaks. Finally, the nuclei were counterstained with DAPI to facilitate the identification of apoptotic cells under a fluorescence microscope.

For paraffin-embedded xenograft tumor sections, a chromogenic TUNEL assay was performed using an equivalent DAB-based system. After deparaffinization, rehydration, and proteinase K treatment, sections were incubated with TdT enzyme and dUTP, followed by peroxidase-conjugated detection and DAB substrate to generate brown staining in apoptotic nuclei. Sections were counterstained with hematoxylin and examined under a light microscope. Apoptotic index was calculated as the percentage of DAB-positive cells in randomly selected fields.

### Mouse xenograft tumor model

Nude mice (*n* = 12, 5–6 weeks old, weight 25–30 g) were procured from SIPPR-BK Laboratory Animal Company, Shanghai. A total of 5 × 10^6^ cells were injected into the legs of each nude mouse. One week after inoculation, the tumor size was measured by caliper every 7 days, and the subcutaneous tumor tissue volume (mm^3^) of nude mice in each group was calculated according to the formula V = (ab2 × 0.5) (a was the long diameter, b was the short diameter). After 3 weeks of recording, all the mice were sacrificed, and the subcutaneous tumor tissues of the mice were stripped, the long and short diameters of the tumor tissue were measured by caliper to calculate the volume (mm^3^), and the weight (mg) was weighed by balance.

### MeDIP-qPCR

The MeDIP-PCR assay was performed using the MethylCollector Ultra Kit (Active Motif, Cat# 55005). Initially, genomic DNA was extracted and sheared via sonication. The fragmented DNA was then subjected to immunoprecipitation using a 5-methylcytosine antibody provided in the kit. Following immunoprecipitation, methylated DNA fragments were captured, washed, and eluted. The purified DNA was subsequently amplified by PCR to analyze methylation patterns at MRPS17 promoters using the following primer pair:

Forward primer: 5’-GCGCGTTAGGAAACCAGAGG-3’.

Reverse primer: 5’-GCTGGGCCGGGGAGGGGGAC-3’.

### MRPS17-related gene enrichment analysis

In LUAD, correlation analysis between MRPS17 and all other genes was performed, Genes with *p* < 0.05 were selected and ranked according to correlation coefficient, and the top50 gene with the largest positive correlation coefficient and the top50 gene with the largest negative correlation coefficient were selected, along with MRPS17 itself, to draw heat maps together. Heatmap samples were ordered from left to right in order of MRPS17 gene expression from high to low, colors represent homogenized gene expression, with more red colors showing higher expression and more blue colors showing lower expression. Heat maps were drawn using the R package pheatmap, and correlations were calculated by the Pearson method. Then, GSEA was performed to predict the mechanism of action of MRPS17 in LUAD. The GSEA gene set used here is the newly updated HALLMARK pathway dataset of the MsigDB database, which contains 50 star pathways. First, LUAD was scored by GSEA, the correlation between gene expression and pathway score was calculated, and a correlation map was generated. Subsequently, GSEA of the KEGG database of MRPS17 in LUAD was performed with the R package “clusterprofiler”.

### Statistical analysis

Data analysis and mapping were performed using GraphPad Prism (version 9.0). The images were analyzed using Fiji software. A T test, Kruskal‒Wallis test and Wilcox rank sum test were used to compare the statistical indicators. The KM test was used to estimate survival. Differences in survival rates were compared using the log-rank test. *P* < 0.05 was considered statistically significant in this investigation.

## Results

### MRPS17 is significantly upregulated in LUAD through bioinformatics analysis

**Fig. 1:**
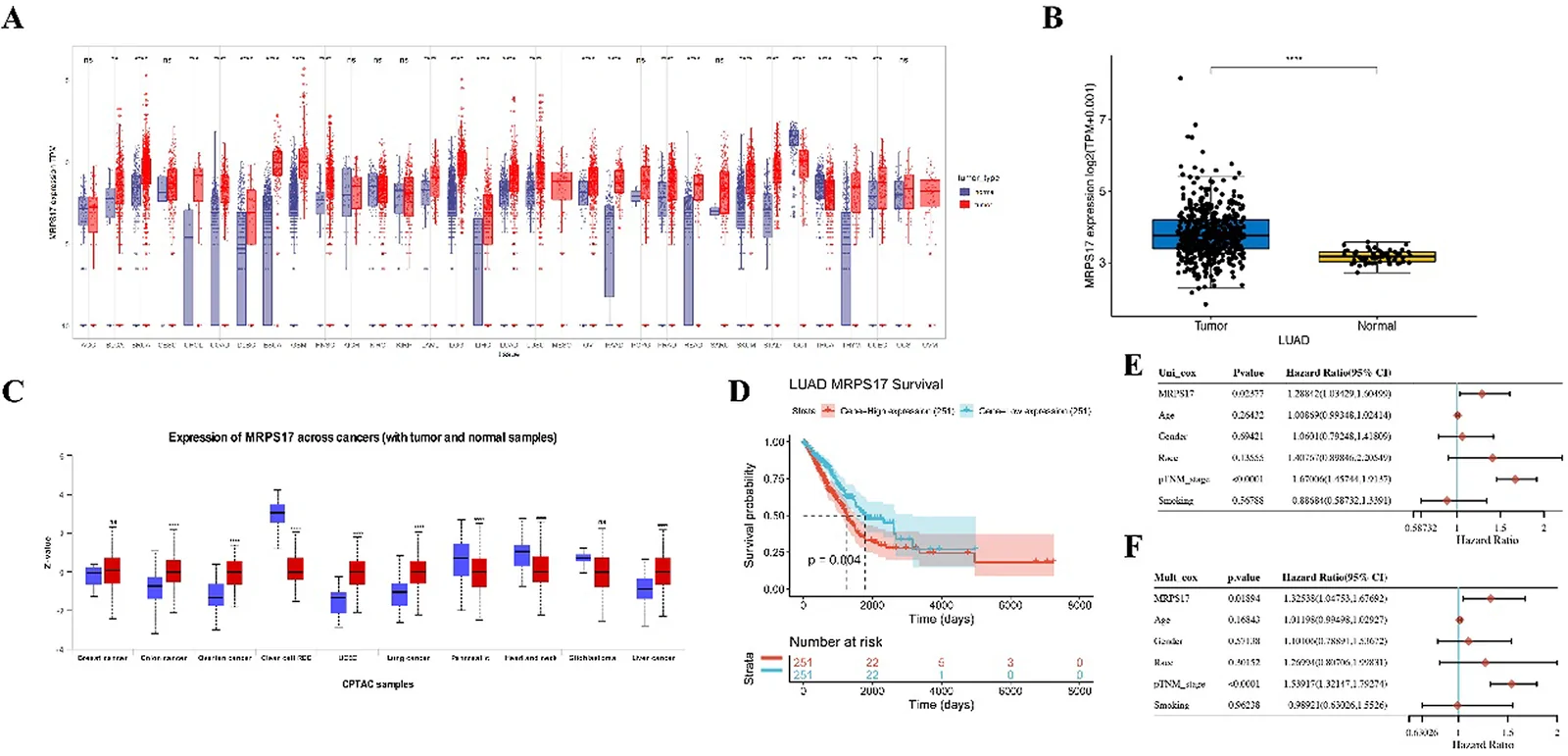
Overexpression of MRPS17 in LUAD through bioinformatics analysis. **A** The MRPS17 expression level in 33 types from the GTEx database and TCGA database; The colour refers to the tumour (red) or normal (blue). **B** The mRNA expression of MRPS17 in LUAD; **C** MRPS17 protein expression in 21 tumour cell types from the CPTAC database; **D** Kaplan‒Meier curves show the association between MRPS17 expression and OS in LUAD; Statistical significance was assessed using the log-rank test. **E**, **F** Univariate (**E**) and multivariate (**F**) Cox regression were performed in LUAD. *P < 0.05; **P < 0.01; ***P < 0.001; ****P < 0.0001

First, the expression of MRPS17 in 33 different human cancers was analyzed using data from the TCGA and GTEx datasets. Figure 1A shows that MRPS17 was highly expressed in 21 cancers. Specifically, Fig. 1B demonstrates significant overexpression of MRPS17 in LUAD tissues compared to normal tissues. To further explore MRPS17 expression across cancers, we performed a proteogenomic analysis using the CPTAC database, which includes data on total protein and phosphorylated protein levels. Our analysis revealed elevated MRPS17 protein expression in colon, ovarian, endometrial, lung, and liver cancer tissues compared to normal tissues (Fig. 1C). Clinically, LUAD patients with high MRPS17 displayed shorter survival time than those with low MRPS17 (Fig. 1D). We then performed univariate Cox regression analysis to examine the associations of MRPS17, age, sex, race, TNM stage, and smoking status with OS in LUAD patients. The results indicated that MRPS17 (*P* = 0.0237, HR = 1.28842) and TNM stage (*P* < 0.0001, HR = 1.67006) were significant factors affecting OS, while age, sex, race, and smoking status were not significantly correlated with OS (Fig. 1E). To ensure all relevant factors were considered, we included all variables in a multivariate Cox regression model. The multivariate analysis confirmed that MRPS17 expression (*P* = 0.01894, HR = 1.32538) and TNM stage (*P* < 0.0001, HR = 1.53917) were significantly associated with OS, whereas age, sex, race, and smoking status were not (Fig. 1F). These findings suggest that MRPS17 expression and TNM stage are key determinants of OS in LUAD patients. The combined gene and protein expression analyses strongly support the role of MRPS17 as a potential oncogene relevant to multiple cancer types.

### MRPS17 exhibits high expression in LUAD tissues and cells

**Fig. 2:**
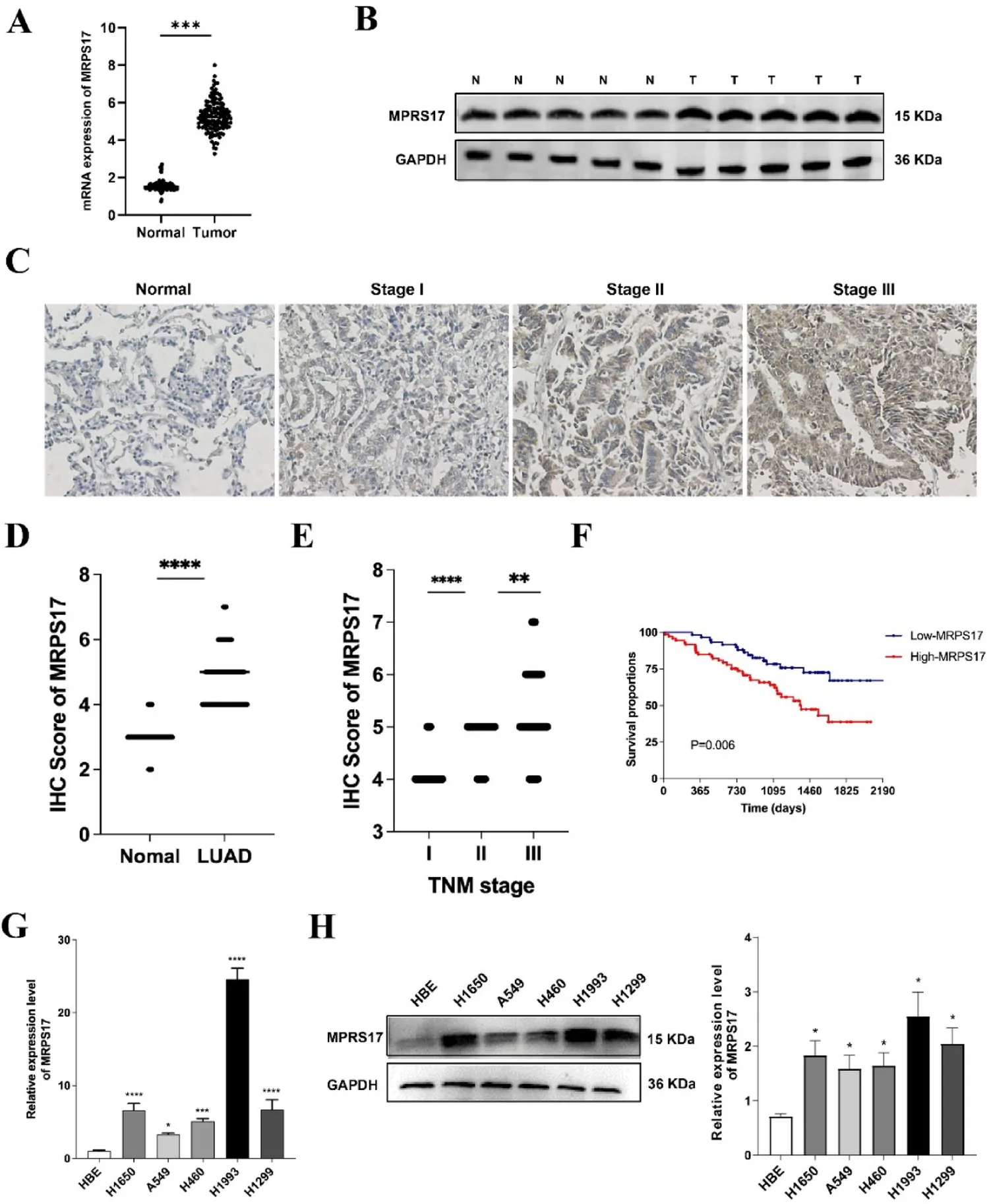
Upregulation of MRPS17 in LUAD tissues and cells. **A**, **B** mRNA (**A**) and protein (**B**) expression of MRPS17 in paired LUAD and adjacent tissues (*n* = 132); **C** Representative IHC images showing MRPS17 expression in LUAD and adjacent tissues (*n* = 132); **D** IHC score of MRPS17 in LUAD and adjacent tissues (*n* = 132); **E** IHC score of MRPS17 according to TNM stage in LUAD tissues (*n* = 132); **F** Survival analysis of LUAD patients stratified by median MRPS17 expression in LUAD tissues (*n* = 132); **G**, **H** mRNA (**G**) and protein (**H**) expression of MRPS17 in A549, H1993, H1299, H460, H1650 and HBE cells (*n* = 3 independent experiments). Quantitative experimental data are presented as mean ± SD unless otherwise indicated. Paired comparisons between LUAD and adjacent tissues in (**A**, **B**) were analyzed using paired Student’s t-test. IHC scores in (**D**) were analyzed using the Wilcoxon signed-rank test. IHC scores among different TNM stages in (**E**) were analyzed using the Kruskal–Wallis test with Dunn’s post hoc test. Survival data in (**F**) were analyzed using the Kaplan–Meier method and compared by the log-rank test. Multiple-group comparisons in (**G**, **H**) were analyzed using one-way ANOVA. *p* < 0.05 was considered statistically significant

To determine the expression level of MRPS17 in LUAD, we collected 132 pairs of LUAD and adjacent normal tissues for qRT-PCR and WB analysis. Our results demonstrated significant upregulation of MRPS17 in LUAD tissues (Fig. 2A, B). Subsequent IHC analysis further confirmed that MRPS17 expression was substantially higher in LUAD tissues compared to normal lung tissues (Fig. 2C, D). Moreover, we observed that MRPS17 protein levels increased with advancing TNM stage (Fig. 2E). Clinical correlation analysis revealed that high MRPS17 expression was significantly associated with advanced TNM stage and lymph node metastasis (*P* < 0.001). Conversely, there was no significant correlation between MRPS17 expression and patient sex, age, or tumor size (Table 1). In line with these findings, LUAD patients with elevated MRPS17 levels exhibited shorter overall survival (OS) compared to those with lower expression (Fig. 2F). Additionally, analysis of MRPS17 expression in NSCLC cell lines (A549, H1993, H1299, H460, H1650) and normal bronchial epithelial (HBE) cells revealed elevated MRPS17 levels across all tested NSCLC lines (Fig. 2G, H). These results are consistent with public database outcomes and underscore MRPS17’s significant overexpression in LUAD, correlating with poor prognosis.

**Table 1:** Correlation analysis between the expression level of MRPS17 and clinical pathological parameters

Parameters		All cases (*n* = 132)	MRPS17 Expression		*P* value
			Low (*n* = 59)	High (*n* = 73)	
Age (years)	≥ 60	59	31	28	0.204
	< 60	73	28	45	
Gender	Male	50	20	30	0.674
	Female	82	39	43	
Tumor stage	I	50	44	6	< 0.001
	II	20	5	15	0.009
	III	62	10	52	
Tumor size	≥ 3 cm	85	36	49	0.116
	< 3 cm	47	23	24	
Lymphnode metastasis	Present	74	17	57	< 0.001
	Absent	58	42	16	

### MRPS17 stimulates the LUAD tumorigenicity in vitro

**Fig. 3:**
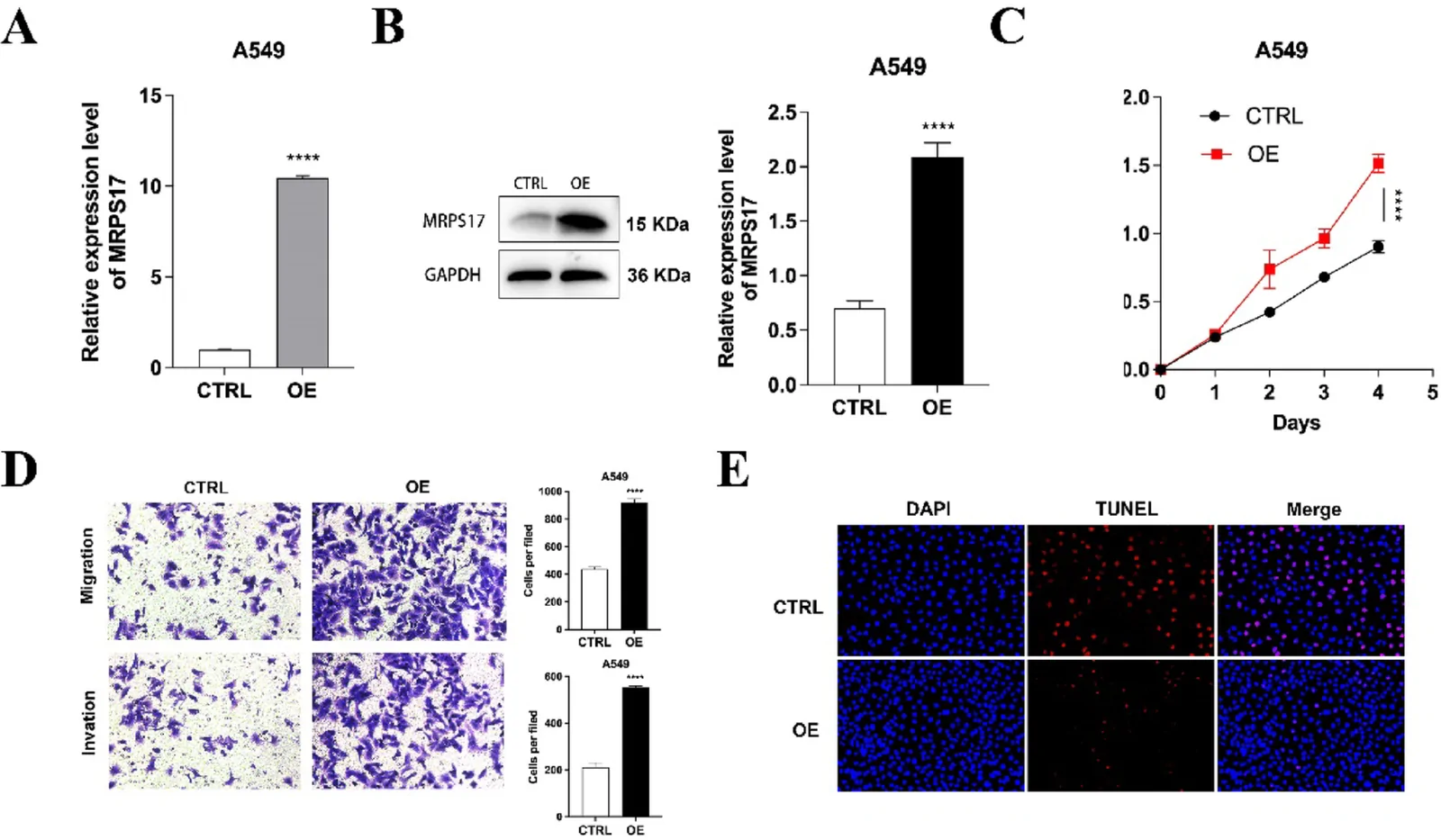
MRPS17 overexpression induces LUAD tumorigenicity in vitro. **A**, **B** The efficiency of MRPS17 overexpression in A549 cells was detected by qRT-PCR (**A**) and Western blot (**B**); **C** The effect of MRPS17 on the proliferative capacity of LUAD cells was detected by CCK-8 assay; **D** The effect of MRPS17 on the migration and invasion abilities of LUAD cells was detected by transwell assay; **E** The effect of MRPS17 on apoptosis in LUAD cells was detected by TUNEL assay (20×). All data are presented as mean ± SD from *n* = 3 independent experiments. Comparisons between two groups in (**A**, **B**, **D**, **E**) were analyzed using an unpaired two-tailed Student’s t-test. The CCK-8 assay in (**C**), involving both group and time factors, was analyzed using two-way ANOVA. *p* < 0.05 was considered statistically significant

First, we generated MRPS17-overexpressing A549 cell lines using an MRPS17 overexpression vector, with the upregulation confirmed by qRT-PCR and WB analyses (Fig. 3A, B). CCK-8 assay results indicated that increased MRPS17 expression significantly enhanced the proliferation of A549 cells (Fig. 3C). Similarly, enforced expression of MRPS17 significantly enhanced both the invasiveness and migration capabilities of these cells, as demonstrated by Transwell assays (Fig. 3D). Additionally, TUNEL assays revealed that MRPS17 overexpression significantly reduced the number of apoptotic cells (Fig. 3E). Collectively, these findings underscore the oncogenic role of MRPS17 in LUAD.

**Fig. 4:**
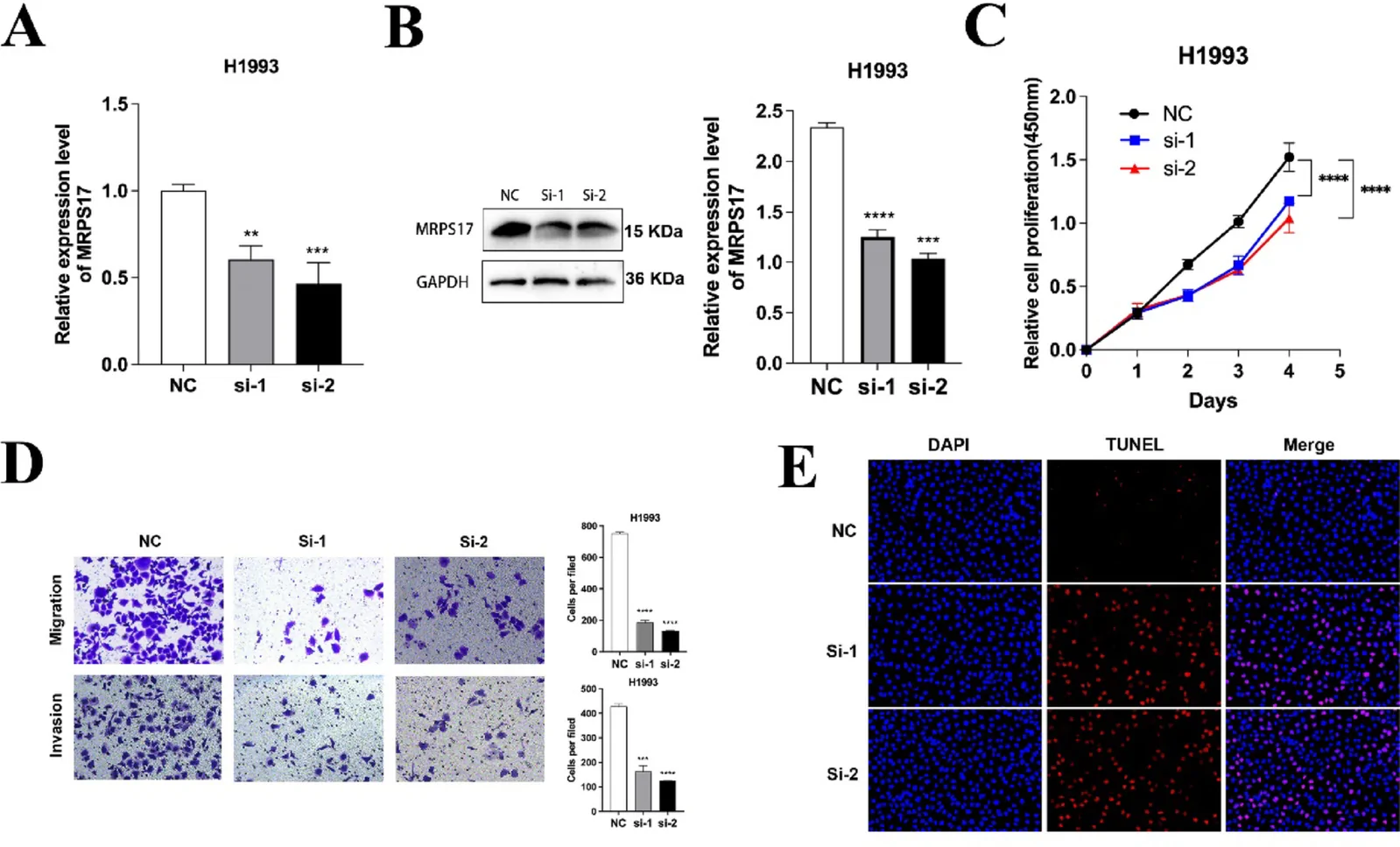
MRPS17 deletion restrains LUAD malignancy in vitro. **A**, **B** The efficiency of MRPS17 inhibition in H1993 cells detected by qRT‒PCR (**A**) and Western blot (**B**); **C** The effect of MRPS17 on the proliferative capacity of LUAD cells detected by CCK-8 assay. **D** The effect of MRPS17 on the migration and invasion ability of LUAD cells detected by transwell assay. **E** The effect of MRPS17 on the apoptosis ability of LUAD cells detected by TUNEL assay(20X). All data are presented as mean ± SD from *n* = 3 independent experiments. **A**, **B**, **D**, **E** were analyzed using the Student’s t-test. **C** was analyzed using two-way ANOVA. *p* < 0.05 was considered statistically significant

Subsequently, due to its relatively high MRPS17 expression, we chose H1993 cells to generate MRPS17-knockdown cell lines. The knockdown efficiency was confirmed by qRT-PCR and WB (Fig. 4A, B). Depletion of MRPS17 led to a significant reduction in cell viability, as evidenced by the results (Fig. 4C). Transwell assays further demonstrated that downregulation of MRPS17 markedly reduced the invasive and migratory abilities of these cells (Fig. 4D). Additionally, MRPS17 silencing resulted in a higher number of apoptotic cells in H1993 compared to control cells (Fig. 4E). These results indicate that MRPS17 knockdown inhibits the aggressive phenotype of LUAD cells.

### MRPS17 promotes LUAD carcinogenicity in vivo

**Fig. 5:**
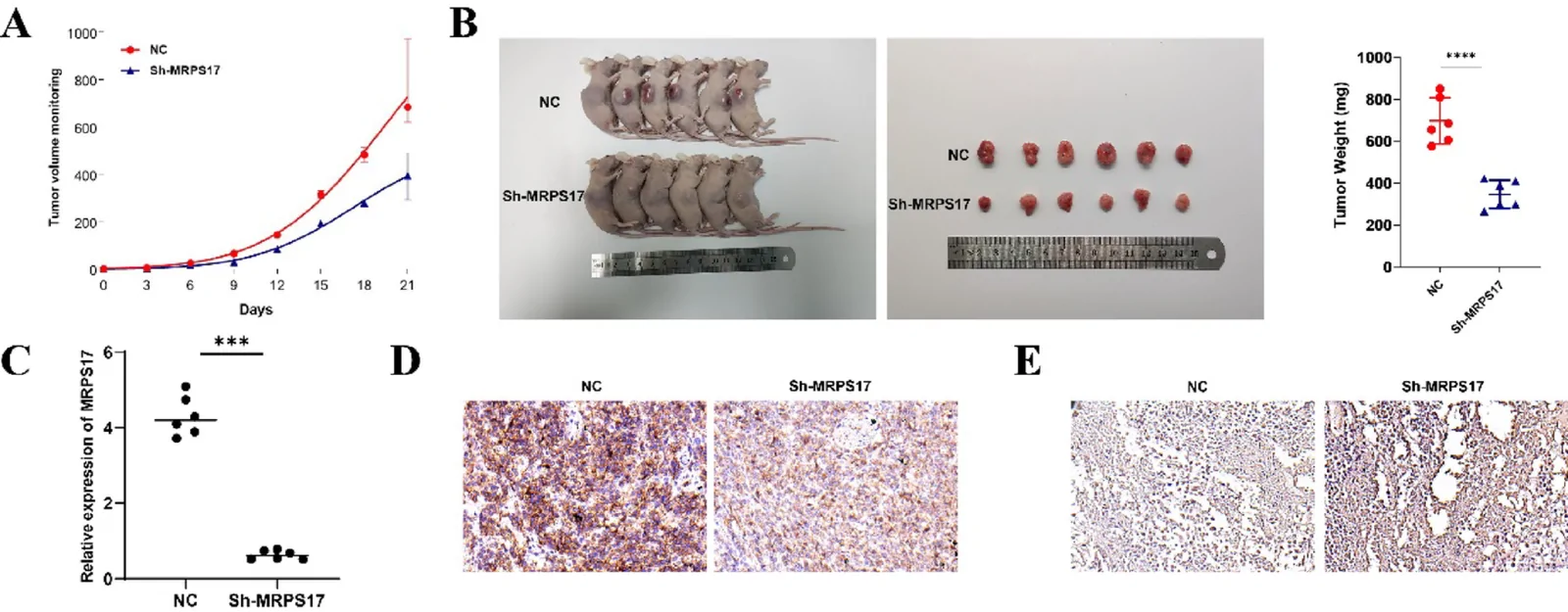
MRPS17 promotes LUAD carcinogenicity in vivo. **A** Xenograft tumor growth in nude mice injected with H1993 cells with MRPS17-deficient; **B** The volume of tumors in nude mice injected with H1993 cells with MRPS17-deficient; **C**, **D** mRNA (**C**) and protein (**D**) expression of MRPS17 in the tumors of nude mice; **E** The effect of MRPS17 on the apoptosis ability of tumors in nude mice detected by TUNEL assay (20X). All data are presented as mean ± SD from *n* = 5 mice per group. **A**, **B**, **C**, **D**, **E** were analyzed using the Student’s t-test for two-group comparisons and two-way ANOVA for multiple time-point comparisons. *p* < 0.05 was considered statistically significant

To investigate the role of MRPS17 in LUAD tumorigenesis in vivo, we performed xenograft experiments using H1993 cells with MRPS17 knockdown (Sh-MRPS17) and control cells (NC). The growth of xenograft tumors in nude mice was monitored over three weeks. The results showed that tumors formed by Sh-MRPS17 cells exhibited significantly reduced growth compared to the control group (Fig. 5A). Furthermore, the volume and weight of tumors derived from Sh-MRPS17 cells were significantly lower than those from control cells, indicating that MRPS17 knockdown inhibits tumor growth in vivo (Fig. 5B). qRT-PCR and IHC analyses confirmed that MRPS17 expression at both mRNA and protein levels was effectively reduced in the tumors from the Sh-MRPS17 group compared to the NC group (Fig. 5C, D). Additionally, TUNEL assays demonstrated an increase in apoptotic cells in tumors with MRPS17 knockdown, suggesting that MRPS17 silencing enhances apoptosis in LUAD tumors (Fig. 5E). These above results demonstrate that MRPS17 functions as an oncogene in LUAD.

### TET1 regulates MRPS17 expression in LUAD

**Fig. 6:**
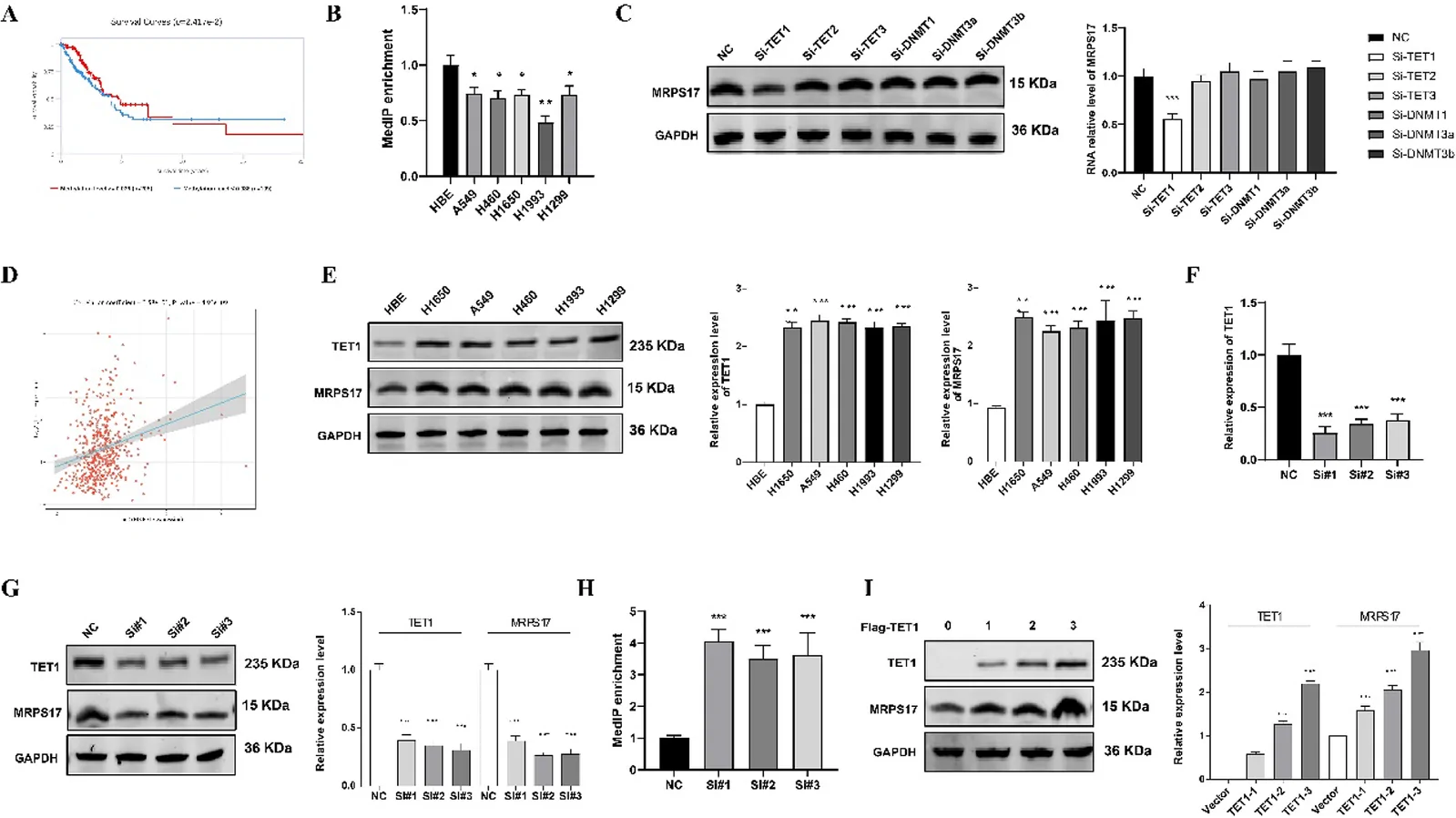
TET1 regulates MRPS17 expression in LUAD. **A **Survival analysis comparing high and low methylation level of MRPS17; **B** MedIP analysis for the methylation level of MRPS17 promoter in HBE, A549, H460, H1650, H1993 and H1299 cells; **C** MRPS17 protein levels in H1993 cells transfected with different DNA methylase (DNMT1, DNMT3a and DNMT3b) and demethylase (TET1, TET2, TET3) knockdown; **D** The relationship between TET1 and MRPS17 in TCGA LUAD cohort; **E** The protein expression of TET1 and MRPS17 in HBE, A549, H460, H1650, H1993 and H1299 cells; **F** mRNA expression of TET1 in TET1-deficient H1993 cells. **G** Protein levels of TET1 and MRPS17 in TET1-deficient H1993 cells; **H** MedIP analysis for the methylation level of MRPS17 promoter in TET1-deficient H1993 cells; **I** Protein levels of TET1 and MRPS17 in H1993 cells transfected with indicated amounts of Flag-TET1-expressing plasmids. Experimental data are presented as mean ± SD from *n* = 3 independent experiments. Survival analysis in (**A**) was performed using the log-rank test. **D** was analyzed using Pearson correlation analysis. Comparisons among multiple experimental groups in (**B**, **C**, **E**, **I**) were analyzed using one-way ANOVA, while comparisons between two groups in (**F**, **H**) were analyzed using Student’s t-test. *p* < 0.05 was considered statistically significant

By the analysis of EWAS Data Hub, survival analysis comparing high and low methylation levels of MRPS17 showed that higher methylation levels were associated with poorer survival outcomes (Fig. 6A). We performed MedIP analysis to assess the methylation level of the MRPS17 promoter in different cell lines, including HBE, A549, H460, H1650, H1993, and H1299 cells. The results indicated significantly lower methylation in the LUAD cell lines compared to HBE cells (Fig. 6B). Next, we analyzed MRPS17 protein levels in H1993 cells transfected with siRNAs targeting various DNA methylases (DNMT1, DNMT3a, and DNMT3b) and demethylases (TET1, TET2, and TET3). Knockdown of TET1 resulted in a significant reduction in MRPS17 protein levels, while knockdown of DNMTs did not show a significant effect (Fig. 6C). An intriguing finding from TCGA database analysis was a notable correlation linking TET1 and MRPS17 expression in LUAD specimens (Fig. 6D). We further examined the protein expression levels of TET1 and MRPS17 across different cell lines, including HBE, A549, H460, H1650, H1993, and H1299. The results showed a correlation between TET1 and MRPS17 expression levels, with higher expression in cancer cell lines compared to normal HBE cells (Fig. 6E). In TET1-deficient H1993 cells, qRT-PCR analysis confirmed a significant reduction in TET1 mRNA levels (Fig. 6F), which was accompanied by decreased MRPS17 mRNA and protein levels (Fig. 6G). MedIP analysis further confirmed an increase in MRPS17 promoter methylation in TET1-deficient cells (Fig. 6H). Finally, we examined the effect of overexpressing TET1 on MRPS17 expression by transfecting H1993 cells with increasing amounts of Flag-TET1-expressing plasmids. The results showed that TET1 overexpression led to a dose-dependent increase in MRPS17 protein levels (Fig. 6I). To summarise, the increase of TET1 in LUAD is attributed to the upregulation of MRPS17.

### TET1 promotes LUAD tumorigenesis through regulating MRPS17

**Fig. 7:**
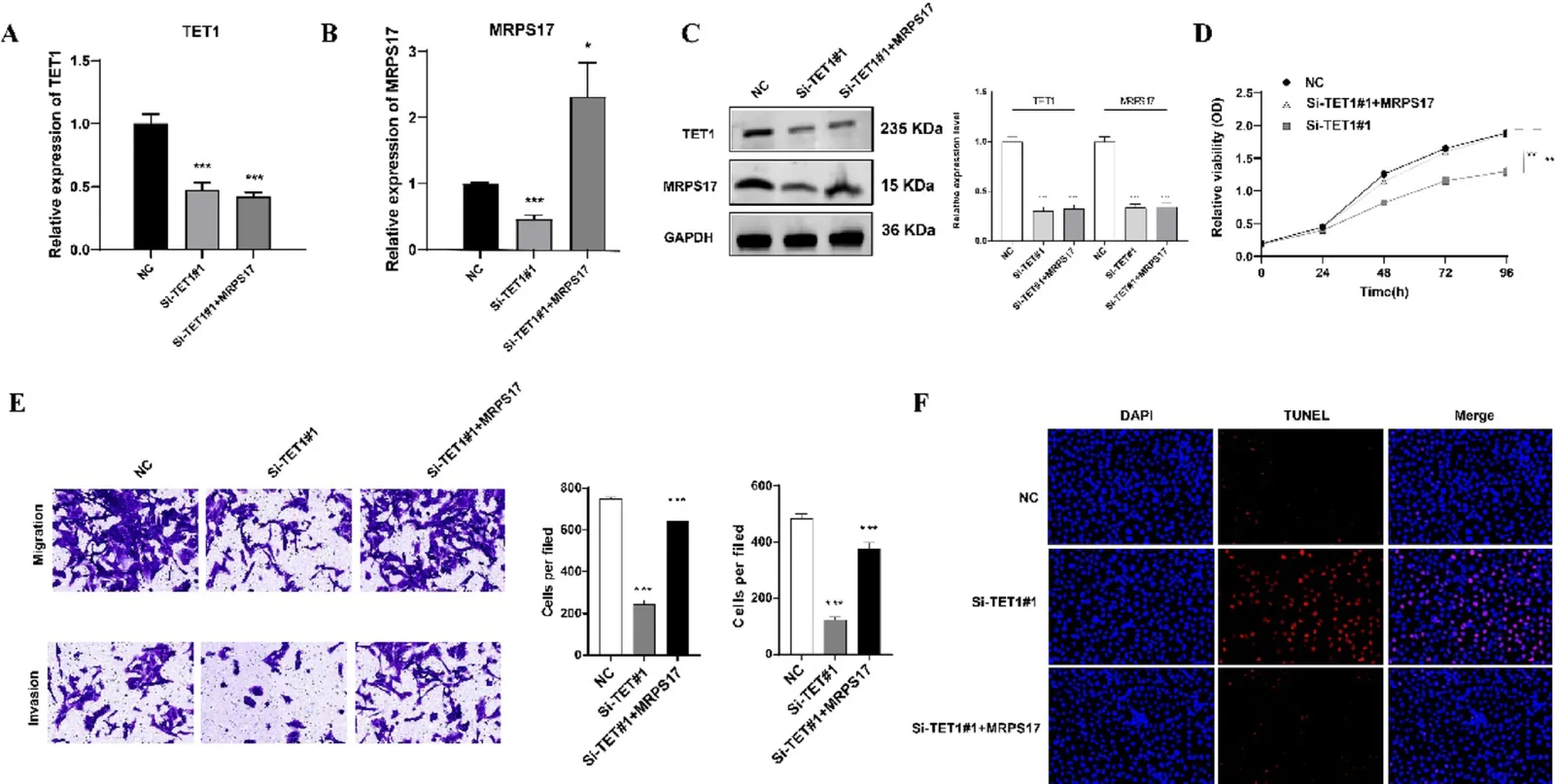
TET1 promotes LUAD oncogenesis through regulating MRPS17. **A** mRNA expression of TET1 in H1993 cells with TET1-deficient or MRPS17-overexpressing; **B** mRNA expression of MRPS17 in H1993 cells with TET1-deficient or MRPS17-overexpressing; **C** Protein expression of TET1 and MRPS17 in H1993 cells with TET1-deficient or MRPS17-overexpressing; **D** CCK-8 staining in H1993 cells with TET1-deficient and MRPS17-upregulated. **E** Transwell assays in H1993 cells with TET1-deficient or MRPS17-overexpressing; **F** TUNEL assays in H1993 cells with TET1-deficient or MRPS17-overexpressing. All data are presented as mean ± SD from *n* = 3 independent experiments. **A**, **B**, **C**, **E**, **F** were analyzed using one-way ANOVA. **D** was analyzed using two-way ANOVA. *p* < 0.05 was considered statistically significant

To investigate the role of TET1 in regulating MRPS17 and its impact on LUAD oncogenesis, we conducted experiments using H1993 cells subjected to TET1 knockdown (Si-TET1#1) and concurrent MRPS17 overexpression (Si-TET1#1 + MRPS17). qRT-PCR analysis revealed that TET1 mRNA levels were significantly reduced in Si-TET1#1 cells compared to NC group. Despite MRPS17 overexpression, TET1 mRNA levels in Si-TET1#1 + MRPS17 cells remained as low as in Si-TET1#1 cells (Fig. 7A). Conversely, while MRPS17 mRNA levels decreased in Si-TET1#1 cells, they increased significantly when MRPS17 was overexpressed in the context of TET1 knockdown (Fig. 7B). WB analysis confirmed these alterations at the protein level; TET1 protein expression was reduced in Si-TET1#1 cells with a corresponding decrease in MRPS17 protein levels. However, MRPS17 overexpression under TET1 knockdown conditions restored MRPS17 protein levels to near normal (Fig. 7C). CCK-8 assay showed a significant reduction in cell survival in TET1-deficient cells compared to controls. Notably, cell viability improved significantly in Si-TET1#1 + MRPS17 cells compared to Si-TET1#1 cells alone, indicating that MRPS17 overexpression could mitigate the proliferation deficits caused by TET1 knockdown (Fig. 7D). Transwell assays demonstrated that migration and invasion capabilities, significantly impaired in Si-TET1#1 cells, were partially recovered in Si-TET1#1 + MRPS17 cells, suggesting that MRPS17 overexpression compensates for TET1 loss (Fig. 7E). Furthermore, TUNEL assays indicated an increase in apoptotic cells in TET1-deficient cells relative to controls. In contrast, MRPS17 overexpression in TET1-deficient cells reduced apoptosis, suggesting that MRPS17 can counteract the apoptosis induced by TET1 knockdown (Fig. 7F). These findings collectively indicate that TET1 supports LUAD oncogenesis through modulation of MRPS17 expression.

### MRPS17 promotes the malignant development of LUAD by regulating the PI3K-AKT-mTOR signaling pathway

**Fig. 8:**
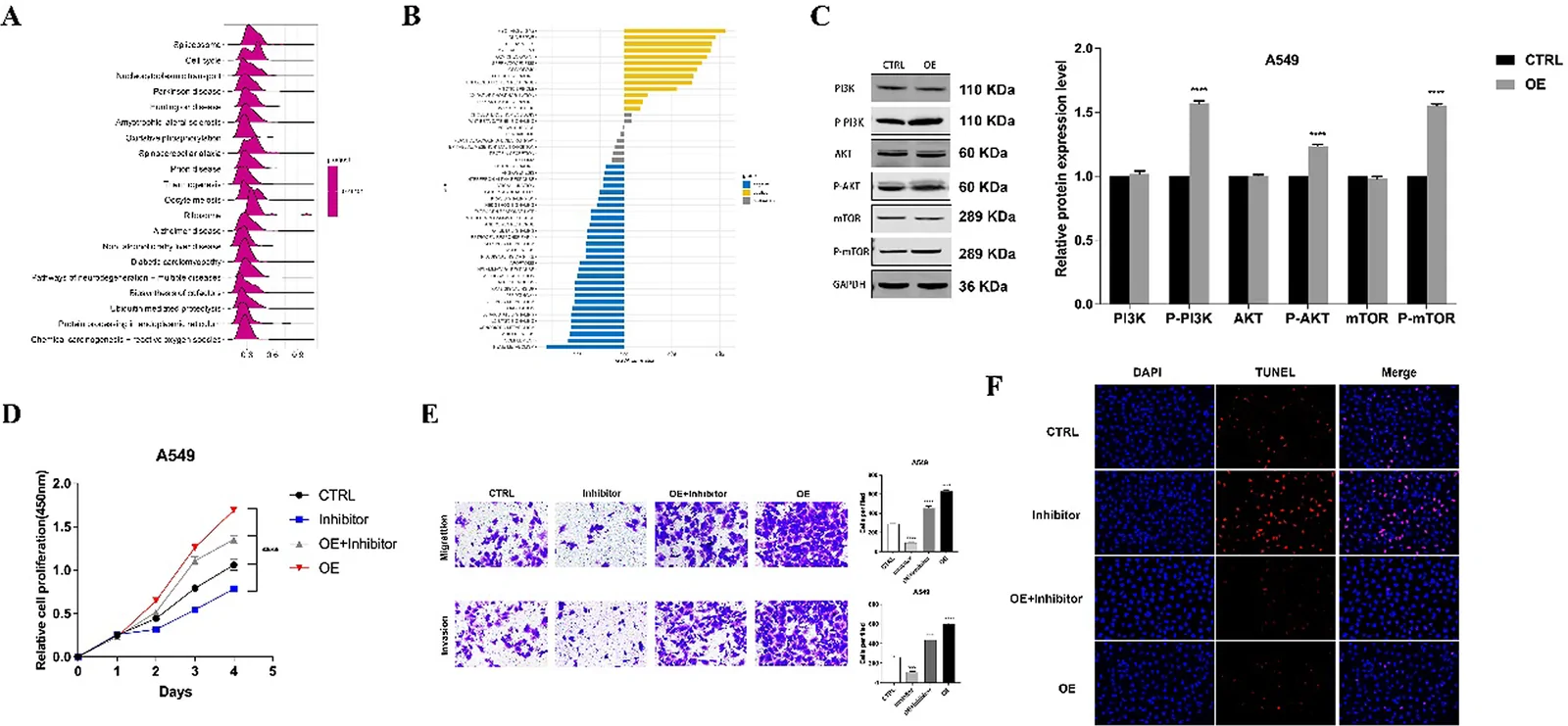
MRPS17 promotes the malignant development of LUAD by regulating the PI3K-AKT-mTOR signalling pathway. **A**, **B** GO (**A**) and KEGG (**B**) analysis of MRPS17 GSEA in LUAD; **C** MRPS17 overexpression promotes P-PI3K, P-AKT and P-mTOR expression; **D** CCK-8 assay showed that MRPS17 promoted the in vitro activity of LUAD cells by regulating the PI3K/AKT/mTOR pathway; **E** Transwell assay results showed that MRPS17 promoted the migration and invasion of LUAD cells by regulating the PI3K/AKT/mTOR pathway; **F** TUNEL assay results showed that MRPS17 inhibited the apoptosis of LUAD cells by regulating the PI3K/AKT/mTOR pathway. LY294002 (PI3K inhibitor) was used at a concentration of 20 µM to block the PI3K pathway. All data are presented as mean ± SD from *n* = 3 independent experiments. **C**, **E**, **F** were analyzed using one-way ANOVA. **D** was analyzed using two-way ANOVA. *p* < 0.05 was considered statistically significant

To elucidate the functions of MRPS17, we performed GO and KEGG analyses through GSEA in LUAD. These analyses revealed significant enrichment of genes associated with MRPS17, particularly in pathways linked to MYC transcription factors, DNA repair, T2F, G2/M checkpoint, spermatogenesis, glycolysis, mTORC1 signaling, protein folding response, mitotic spindle, oxidative phosphorylation, the PI3K/AKT/mTOR signaling pathway, and ultraviolet response (Fig. 8A, B). WB analysis showed that MRPS17 overexpression in A549 cells resulted in increased phosphorylation of PI3K, AKT, and mTOR proteins, indicating activation of the PI3K-AKT-mTOR signaling pathway (Fig. 8C). The CCK-8 assay further demonstrated that MRPS17 overexpression significantly enhanced LUAD cell proliferation. This proliferative effect was markedly reduced by the inhibition of the PI3K-AKT-mTOR pathway, suggesting that MRPS17’s promotion of cell proliferation operates primarily through this pathway (Fig. 8D). Additionally, Transwell assays revealed that MRPS17 overexpression augmented the migration and invasion capabilities of LUAD cells. Inhibition of the PI3K-AKT-mTOR pathway attenuated these effects, underscoring MRPS17’s role in enhancing cell migration and invasion via this signaling route (Fig. 8E). Furthermore, TUNEL assays indicated that MRPS17 overexpression reduced apoptosis in LUAD cells. Conversely, inhibiting the PI3K-AKT-mTOR pathway reversed this anti-apoptotic effect, reinforcing MRPS17’s regulatory influence on apoptosis through this pathway (Fig. 8F). Collectively, these findings suggest that MRPS17 promotes the malignant progression of LUAD by activating the PI3K-AKT-mTOR signaling pathway.

## Discussion

Compared with previous studies linking MRPS17 to prognosis in NSCLC and to PI3K/AKT signaling in gastric cancer, the present study identifies an unrecognized epigenetic mechanism underlying MRPS17 dysregulation in LUAD. Specifically, we show that TET1-mediated DNA demethylation is associated with MRPS17 upregulation and that the TET1/MRPS17 axis promotes malignant phenotypes of LUAD cells. To our knowledge, this is the first study to connect MRPS17 overexpression in LUAD with an upstream demethylation mechanism and a downstream PI3K-AKT-mTOR oncogenic program. Another major strength of this work is the integration of public multi-omics datasets with institutional clinical specimens and functional validation. By combining TCGA/GTEx/CPTAC analyses, a 132-patient LUAD cohort, in vitro gain- and loss-of-function assays, xenograft experiments, and rescue studies, we provide convergent evidence that MRPS17 is not merely a prognostic correlate but a functional driver of LUAD progression. These data extend prior descriptive observations and support MRPS17 as a mechanistically relevant biomarker and potential therapeutic target in LUAD.

We conducted a comprehensive analysis of the expression level and clinical significance of MRPS17 across cancers, especially LUAD, by using clinical samples and basic experiments for data analysis and subsequent validation. The availability of a large amount of genomic data for free from public repositories, such as the TCGA, GTEx, and CPTAC, has enabled researchers to analyze large datasets of diseases, such as those for tumors, and to obtain valuable information (Mounir et al. 2019). Our analysis revealed that MRPS17 was significantly expressed in 21 cancers and was substantially linked with the prognosis of LUAD.

We obtained many samples from public databases for analysis and then combined them with data from our center and basic experiments for validation. This combination of public data and in vivo and in vitro data for multilevel validation will provide a stronger scientific basis for our results and a more comprehensive validation of our study (Alnafakh et al. 2021, Heo et al. 2020, Jiang et al. 2022). Our study’s findings showed that MRPS17 was much more highly expressed in all NSCLC cell lines than in the HBE cells. Additionally, our analysis of clinical samples through immunohistochemistry experiments confirmed the abnormally high expression of MRPS17 in LUAD tissues. Additionally, in LUAD patients, MRPS17 expression was adversely linked with OS, indicating a potential function for MRPS17 in the advancement of LUAD. To gain a deeper understanding of the role of MRPS17 in LUAD, we carried out several in vitro tests. The CCK-8 and Transwell assay findings showed that LUAD cell proliferation, motility, and invasiveness were greatly enhanced by MRPS17 overexpression. On the other hand, LUAD cell migration and invasion were significantly inhibited by MRPS17 knockdown. Following this, in vivo investigations were conducted, and the results of the nude mouse xenograft tumor formation test showed that LUAD cell proliferation was accelerated by MRPS17 overexpression. Naturally, MRPS17 overexpression and knockdown cannot be entirely blamed for the changed proliferative, migrating, and invading capacities of LUAD cells, and senescence and apoptosis are a few other factors that may also have an impact. MRPS17 is categorized as a mitochondrial ribosomal protein and belongs to the S17P family of ribosomal proteins (28s). It plays a pivotal role in the oxidative phosphorylation process. MRPS17 may have an impact on the onset and prognosis of gastric cancer (Zhou et al. 2021). According to a study by Konstantinos et al., older NSCLC patients with unusually high expression of MRPS17 had reduced survival rates. This observation further underscores the potential significance of MRPS17 as a prognostic marker in NSCLC, particularly among elderly individuals (Prokopidis et al. 2022). This is consistent with the results obtained in our study and provides additional evidence that MRPS17 promotes the malignant progression of LUAD. In conclusion, MRPS17 is a putative oncogene that is strongly linked to a poor prognosis in patients with NSCLC, and it shows promise as a novel possible biomarker for prognostic prediction in patients with lipodystrophy.

A novel aspect of our study is the identification of TET1 as a regulator of MRPS17 expression through epigenetic modifications. By demonstrating that TET1-mediated demethylation influences MRPS17 expression, we introduce an epigenetic dimension to the regulation of mitochondrial ribosomal proteins, which may be leveraged for therapeutic purposes. The role of TET1 in cancer is a focal point of research due to its critical involvement in epigenetic modifications that regulate gene expression (Alrehaili et al. 2023). TET1 is part of the TET family of enzymes that catalyze the conversion of 5mC to 5hmC. This modification is crucial for the active demethylation process that can alter gene expression patterns and subsequently affect cancer progression. Research has shown that TET1 plays dual roles in cancer, functioning either as a tumor suppressor or an oncogene, depending on the cancer type and the cellular context. It has been linked with various oncogenic pathways, such as MYC and E2F, which are involved in cell cycle regulation, apoptosis, and cellular proliferation. For instance, TET1-mediated demethylation is implicated in activating oncogenic pathways in aggressive prostate cancers, illustrating its role in promoting tumorigenesis under certain conditions (Costa et al. 2023). Furthermore, TET1 influences the cancer genome through its interaction with other epigenetic regulators. For example, TET1 is involved in the remodeling of heterochromatin, a form of tightly packed DNA that is generally transcriptionally inactive. This includes interactions with polycomb repressive complexes, which play significant roles in maintaining stem cell identity and regulating differentiation pathways that are often disrupted in cancer (Hagihara et al. 2021). The complex role of TET1 highlights its potential as a therapeutic target. By influencing TET1 activity, either through upregulation or inhibition, it might be possible to alter the epigenetic landscape of cancer cells, offering new avenues for cancer treatment (Wu et al. 2018). These insights into TET1’s function underscore the importance of epigenetic mechanisms in cancer and provide a strong foundation for further research into its therapeutic potential.

Next, to investigate the biological processes of LUAD, we used GSEA. Our analyses indicated that MRPS17 may influence the etiology or pathogenesis of LUAD through the PI3K/AKT/mTOR pathway. Numerous investigations have demonstrated that PI3K/AKT is a cancer-promoting pathway (Yu et al. 2022, Zhang et al. 2017, Fang et al. 2016, Gao and Wang 2019, Golob-Schwarzl et al. 2017, Wang et al. 2019, Qian et al. 2017). Zhou et al. reported that MRPS17 promotes gastric cancer development through the PI3K/AKT signaling pathway (Zhou et al. 2021). Chen et al. showed that in LUAD, CCAT1/FABP5 stimulates tumor growth via the PI3K/AKT/mTOR signaling pathway (Chen et al. 2021). Liu et al. reported that the upregulation of CENPM has the potential to advance the progression of lung adenocarcinoma by influencing and modulating the PI3K/AKT/mTOR pathway (Liu et al. 2022). The significance of the PI3K/AKT/mTOR signaling pathway in LUAD is highlighted by these results. Thus, we postulated that MRPS17 activates the PI3K/AKT/mTOR signaling pathway to promote the malignant activity of LUAD. To substantiate our hypothesis, we verified the protein expression level, demonstrating that MRPS17 overexpression heightened the expression of P-PI3K, P-AKT, and P-mTOR. Functional experiments further confirmed our findings, as MRPS17 overexpression strongly mitigated the inhibitory effects of PI3K/AKT/mTOR inhibitors on LUAD cell proliferation, migration, and invasion. All things considered, the study’s findings point to MRPS17’s potential role in the malignant development of LUAD by stimulating the PI3K/AKT/mTOR signaling pathway, which in turn stimulates cell invasion, migration, and proliferation.

GSEA also revealed that MRPS17 was positively correlated with c-Myc and negatively correlated with p53. c-Myc is a transcriptional regulator encoded by the proto-oncogene MYC and plays an important role in cell metabolism and proliferation (Miller et al. 2012). c-Myc can activate many oncogenic pathways leading to malignant transformation and can also be activated by many oncogenic pathways to drive multiple synthetic functions to achieve unlimited cell proliferation while inhibiting the expression of antiproliferative genes (Miller et al. 2012). p53 is a tumor suppressor gene, and its elevated expression can inhibit cell proliferation (Ping et al. 2024). Studies have shown that SETDB1 induces the Warburg effect in the cytoplasm through the c-Myc-LDHA axis and promotes the migration and invasion of breast cancer cells (Yang et al. 2024). The expression of c-Myc in lung cancer cell lines was also significantly increased, and downregulation of c-Myc inhibited the proliferation of lung cancer cells. PI3K/AKT and mTor inhibitors can reduce the expression of c-Myc in cells, thereby inhibiting the invasion, migration and epithelial–mesenchymal transition of lung cancer cells, suggesting that c-Myc may be a downstream target of the PI3K/AKT/mTOR pathway and participate in the regulation of lung cancer cell apoptosis induced by phenethyl isothiocyanate (Hu Jun 2020). Studies have shown that c-Myc is a downstream and positive regulator of the PI3K/AKT signaling pathway, but it can negatively regulate P53. Overexpression of VPS33B could regulate the EGFR/PI3K/AKT/c-Myc/P53 signaling pathway, resulting in cell cycle arrest at the G1/S phase. In addition, the tumor suppressor miRNA miR-133a-3p is induced by P53 and directly targets the EGFR/PI3K/AKT/c-Myc/P53 signaling pathway, thereby forming a negative feedback loop (Liang et al. 2019). Foxo1 inhibits MYH9 protein expression by regulating the PI3K/AKT/c-Myc/P53/miR-133a-3p signaling axis in NPC. After c-Myc transfection, the stemness, migration and invasion ability of FOXO1-overexpressing NPC cells were significantly restored. In addition, the addition of an inhibitor of PI3K to FOXO1-overexpressing NPC cells reduced the expression of PI3K/AKT/c-Myc and MYH9 and upregulated P53/miR-133a-3p signaling. In addition, the binding of P53 to the miR-133a-3p promoter was significantly enhanced in NPC cells (Li et al. 2019). Research has shown that leukaemia is driven by increased Myc, and increased Myc is associated with the loss of p53. The p53 protein also indirectly inhibits glycolysis by increasing the expression of inhibitors of the PI3K/Akt pathway (Jaworska et al. 2023). The above studies suggest that c-Myc and p53 may interact with MRPS17 in the PI3K/AKT/mTOR pathway, affecting cell proliferation, migration and invasion.

Overall, this work demonstrated that MRPS17 expression was connected with the prognosis of LUAD and that MRPS17 was aberrantly highly expressed in a variety of malignancies. We established that MRPS17 is abundantly expressed in LUAD tissues and cells, and our in vitro and in vivo studies verified that MRPS17 drives LUAD cell malignant biological behavior. Because overexpression of MRPS17 stimulates the PI3K/AKT/mTOR signaling pathway, which advances the malignant development of LUAD, it can increase the number, migration, and invasion of LUAD cells. The study findings indicate that there may be a place for MRPS17 as a novel biomarker in the treatment and classification of LUAD patients, since overexpression of the gene was found to be substantially connected with a poor prognosis in these patients.

### In conclusion

Our study elucidates a novel epigenetic mechanism by which MRPS17 is regulated and contributes to LUAD progression, underscoring its potential as a target for precision medicine. The modulation of MRPS17 expression via TET1 highlights a promising therapeutic avenue that could lead to the development of novel treatments for LUAD. Continued investigation into the molecular mechanisms governing MRPS17 and their impact on lung adenocarcinoma will be essential for translating these findings into clinical applications.

## Supplementary Information

Below is the link to the electronic supplementary material.

Supplementary Material 1

Supplementary Material 2

## Data Availability

The expression profiles and clinical data of TCGA, GTEx and CCLE in support of this study were obtained from the UCSC XENA website: [https://xenabrowser.net/datapages/](https:/xenabrowser.net/datapages).

## References

[CR30] Alnafakh R, Saretzki G, Midgley A, Flynn J, Kamal AM, Dobson L et al (2021) Aberrant Dyskerin expression is related to proliferation and poor survival in endometrial cancer. Cancers (Basel) 13(2):273 10.3390/cancers13020273 PMC7828388 33450922

[CR33] Alrehaili AA, Gharib AF, Alghamdi SA, Alhazmi A, Al-Shehri SS, Hagag HM et al (2023) Evaluation of TET family gene expression and 5-hydroxymethylcytosine as potential epigenetic markers in non-small cell lung cancer. Vivo 37(1):445–453 10.21873/invivo.13098 PMC9843776 36593050

[CR25] Baughman JM, Nilsson R, Gohil VM, Arlow DH, Gauhar Z, Mootha VK (2009) A computational screen for regulators of oxidative phosphorylation implicates SLIRP in mitochondrial RNA homeostasis. PLoS Genet 5(8):e1000590 10.1371/journal.pgen.1000590 PMC2721412 19680543

[CR44] Chen J, Alduais Y, Zhang K, Zhu X, Chen B (2021) CCAT1/FABP5 promotes tumour progression through mediating fatty acid metabolism and stabilizing PI3K/AKT/mTOR signalling in lung adenocarcinoma. J Cell Mol Med 25(19):9199–9213 10.1111/jcmm.16815 PMC8500980 34431227

[CR6] Chen P, Quan Z, Song X, Gao Z, Yuan K (2022) MDFI is a novel biomarker for poor prognosis in LUAD. Front Oncol 12:1005962 10.3389/fonc.2022.1005962 PMC9589366 36300089

[CR26] Cheong A, Lingutla R, Mager J (2020) Expression analysis of mammalian mitochondrial ribosomal protein genes. Gene Expr Patterns 38:119147 10.1016/j.gep.2020.119147 PMC7726062 32987154

[CR34] Costa P, Sales SLA, Pinheiro DP, Pontes LQ, Maranhão SS, Pessoa C et al (2023) Epigenetic reprogramming in cancer: from diagnosis to treatment. Front Cell Dev Biol 11:1116805 10.3389/fcell.2023.1116805 PMC9974167 36866275

[CR12] Ferro Dos Santos MR, Giuili E, De Koker A, Everaert C, De Preter K (2024) Computational deconvolution of DNA methylation data from mixed DNA samples. Brief Bioinform 25(3):bbae234 10.1093/bib/bbae234 PMC11102637 38762790

[CR39] Fang WL, Huang KH, Lan YT, Lin CH, Chang SC, Chen MH et al (2016) Mutations in PI3K/AKT pathway genes and amplifications of PIK3CA are associated with patterns of recurrence in gastric cancers. Oncotarget 7(5):6201–6220 10.18632/oncotarget.6641 PMC4868750 26701847

[CR40] Gao BB, Wang SX (2019) LncRNA BC200 regulates the cell proliferation and cisplatin resistance in non-small cell lung cancer via PI3K/AKT pathway. Eur Rev Med Pharmacol Sci 23(3):1093–1101 10.26355/eurrev_201902_16999 30779077

[CR41] Golob-Schwarzl N, Krassnig S, Toeglhofer AM, Park YN, Gogg-Kamerer M, Vierlinger K et al (2017) New liver cancer biomarkers: PI3K/AKT/mTOR pathway members and eukaryotic translation initiation factors. Eur J Cancer 83:56–70 10.1016/j.ejca.2017.06.003 28715695

[CR17] Gopisetty G, Thangarajan R (2016) Mammalian mitochondrial ribosomal small subunit (MRPS) genes: a putative role in human disease. Gene 589(1):27–35 10.1016/j.gene.2016.05.008 27170550

[CR35] Hagihara Y, Asada S, Maeda T, Nakano T, Yamaguchi S (2021) Tet1 regulates epigenetic remodeling of the pericentromeric heterochromatin and chromocenter organization in DNA hypomethylated cells. PLoS Genet 17(6):e1009646 10.1371/journal.pgen.1009646 PMC8263065 34166371

[CR23] Ham S, Oh YM, Roh TY (2019) Evaluation and interpretation of transcriptome data underlying heterogeneous chronic obstructive pulmonary disease. Genomics Inf 17(1):e2 10.5808/GI.2019.17.1.e2 PMC6459164 30929403

[CR24] Hanlon SE, Xu Z, Norris DN, Vershon AK (2004) Analysis of the meiotic role of the mitochondrial ribosomal proteins Mrps17 and Mrpl37 in Saccharomyces cerevisiae. Yeast 21(15):1241–1252 10.1002/yea.1174 15543521

[CR31] Heo MJ, Kang SH, Kim YS, Lee JM, Yu J, Kim HR et al (2020) UBC12-mediated SREBP-1 neddylation worsens metastatic tumor prognosis. Int J Cancer 147(9):2550–2563 10.1002/ijc.33113 32449166

[CR49] Hu Jun XL (2020) Phenyl isothiocyanate inhibit the expression of c-Myc and RPS19 in nonsmall cell lung cancer cells via the PI3K/Akt/mTOR pathway. Chin J Cell Biology 42(10):1774–1781 (in Chinese)

[CR9] Imyanitov EN, Iyevleva AG, Levchenko EV (2021) Molecular testing and targeted therapy for non-small cell lung cancer: current status and perspectives. Crit Rev Oncol Hematol 157:103194 10.1016/j.critrevonc.2020.103194 33316418

[CR52] Jaworska M, Szczudło J, Pietrzyk A, Shah J, Trojan SE, Ostrowska B et al (2023) The Warburg effect: a score for many instruments in the concert of cancer and cancer niche cells. Pharmacol Rep 75(4):876–890 10.1007/s43440-023-00504-1 PMC10374743 37332080

[CR32] Jiang L, Zhang Y, Su P, Ma Z, Ye X, Kang W et al (2022) Long non-coding RNA HNF1A-AS1 induces 5-FU resistance of gastric cancer through miR-30b-5p/EIF5A2 pathway. Transl Oncol 18:101351 10.1016/j.tranon.2022.101351 PMC8802127 35092904

[CR21] Jing C, Fu R, Wang C, Li X, Zhang W (2021) MRPL13 act as a novel therapeutic target and could promote cell proliferation in non-small cell lung cancer. Cancer Manag Res 13:5535–5545 10.2147/CMAR.S316428 PMC8285246 34285575

[CR18] Ju YS, Alexandrov LB, Gerstung M, Martincorena I, Nik-Zainal S, Ramakrishna M et al (2014) Origins and functional consequences of somatic mitochondrial DNA mutations in human cancer. Elife 3:e02935 10.7554/eLife.02935 PMC4371858 25271376

[CR20] Kim HJ, Maiti P, Barrientos A (2017) Mitochondrial ribosomes in cancer. Semin Cancer Biol 47:67–81 10.1016/j.semcancer.2017.04.004 PMC5662495 28445780

[CR51] Li Y, Liu X, Lin X, Zhao M, Xiao Y, Liu C et al (2019) Chemical compound cinobufotalin potently induces FOXO1-stimulated cisplatin sensitivity by antagonizing its binding partner MYH9. Signal Transduct Target Ther 4:48 10.1038/s41392-019-0084-3 PMC6861228 31754475

[CR50] Liang Z, Liu Z, Cheng C, Wang H, Deng X, Liu J et al (2019) VPS33B interacts with NESG1 to modulate EGFR/PI3K/AKT/c-Myc/P53/miR-133a-3p signaling and induce 5-fluorouracil sensitivity in nasopharyngeal carcinoma. Cell Death Dis 10(4):305 10.1038/s41419-019-1457-9 PMC6447525 30944308

[CR45] Liu C, Wang Y, Dao Y, Wang S, Hou F, Yang Z et al (2022) Upregulation of CENPM facilitates lung adenocarcinoma progression via PI3K/AKT/mTOR signaling pathway. Acta Biochim Biophys Sin (Shanghai) 54(1):99–112 10.3724/abbs.2021013 PMC9909302 35130633

[CR5] Liu Y, Zhang H, Zhang W, Xiang L, Yin Z, Xu H et al (2022) circ_0004140 promotes LUAD tumor progression and immune resistance through circ_0004140/miR-1184/CCL22 axis. Cell Death Discov 8(1):181 10.1038/s41420-022-00983-w PMC8993797 35396377

[CR46] Miller DM, Thomas SD, Islam A, Muench D, Sedoris K (2012) c-Myc and cancer metabolism. Clin Cancer Res 18(20):5546–5553 10.1158/1078-0432.CCR-12-0977 PMC3505847 23071356

[CR29] Mounir M, Lucchetta M, Silva TC, Olsen C, Bontempi G, Chen X et al (2019) New functionalities in the TCGAbiolinks package for the study and integration of cancer data from GDC and GTEx. PLoS Comput Biol 15(3):e1006701 10.1371/journal.pcbi.1006701 PMC6420023 30835723

[CR2] Naranjo S, Cabana CM, LaFave LM, Romero R, Shanahan SL, Bhutkar A et al (2022) Modeling diverse genetic subtypes of lung adenocarcinoma with a next-generation alveolar type 2 organoid platform. Genes Dev 36(15–16):936–949 10.1101/gad.349659.122 PMC9575694 36175034

[CR13] Oladipo EK, Olufemi SE, Adediran DA, Adejumo IO, Jimah EM, Oloke JK et al (2024) Epigenetic modifications in solid tumor metastasis in people of African ancestry. Front Oncol 14:1325614 10.3389/fonc.2024.1325614 PMC10915648 38450190

[CR11] Passiglia F, Malapelle U, Normanno N, Pinto C (2022) Optimizing diagnosis and treatment of EGFR exon 20 insertions mutant NSCLC. Cancer Treat Rev 109:102438 10.1016/j.ctrv.2022.102438 35882108

[CR47] Ping N, Hara-Kuge S, Yagi Y, Kazama T, Nakamura T (2024) Translational enhancement of target endogenous mRNA in mammalian cells using programmable RNA-binding pentatricopeptide repeat proteins. Sci Rep 14(1):251 10.1038/s41598-023-50776-z PMC10762265 38167853

[CR16] Pires SF, de Barros JS, da Costa SS, de Oliveira Scliar M, Van Helvoort Lengert A, Boldrini É et al (2023) DNA methylation patterns suggest the involvement of DNMT3B and TET1 in osteosarcoma development. Mol Genet Genomics 298(3):721–733 10.1007/s00438-023-02010-8 37020053

[CR28] Prokopidis K, Giannos P, Witard OC, Peckham D, Ispoglou T (2022) Aberrant mitochondrial homeostasis at the crossroad of musculoskeletal ageing and non-small cell lung cancer. PLoS ONE 17(9):e0273766 10.1371/journal.pone.0273766 PMC9447904 36067173

[CR43] Qian DC, Xiao X, Byun J, Suriawinata AA, Her SC, Amos CI et al (2017) PI3K/Akt/mTOR signaling and plasma membrane proteins are implicated in responsiveness to adjuvant dendritic cell vaccination for metastatic colorectal cancer. Clin Cancer Res 23(2):399–406 10.1158/1078-0432.CCR-16-0623 PMC5611841 27435399

[CR3] Román M, Baraibar I, López I, Nadal E, Rolfo C, Vicent S et al (2018) KRAS oncogene in non-small cell lung cancer: clinical perspectives on the treatment of an old target. Mol Cancer 17(1):33 10.1186/s12943-018-0789-x PMC5817724 29455666

[CR19] Stewart JB, Alaei-Mahabadi B, Sabarinathan R, Samuelsson T, Gorodkin J, Gustafsson CM et al (2015) Simultaneous DNA and RNA mapping of somatic mitochondrial mutations across diverse human cancers. PLoS Genet 11(6):e1005333 10.1371/journal.pgen.1005333 PMC4488357 26125550

[CR1] Sung H, Ferlay J, Siegel RL, Laversanne M, Soerjomataram I, Jemal A et al (2021) Global cancer statistics 2020: GLOBOCAN estimates of incidence and mortality worldwide for 36 cancers in 185 countries. CA Cancer J Clin 71(3):209–249 10.3322/caac.21660 33538338

[CR10] Tan AC, Tan DSW (2022) Targeted therapies for lung cancer patients with oncogenic driver molecular alterations. J Clin Oncol 40(6):611–625 10.1200/JCO.21.01626 34985916

[CR42] Wang L, Li K, Wang C, Shi X, Yang H (2019) miR-107 regulates growth and metastasis of gastric cancer cells via activation of the PI3K-AKT signaling pathway by down-regulating FAT4. Cancer Med 8(11):5264–5273 10.1002/cam4.2396 PMC6718591 31297980

[CR36] Wu X, Li G, Xie R (2018) Decoding the role of TET family dioxygenases in lineage specification. Epigenetics Chromatin 11(1):58 10.1186/s13072-018-0228-7 PMC6172806 30290828

[CR48] Yang W, Wei Y, Wang T, Xu Y, Jin X, Qian H et al (2024) Cytoplasmic localization of SETDB1–induced Warburg effect via c–MYC–LDHA axis enhances migration and invasion in breast carcinoma. Int J Mol Med 53(4):40 10.3892/ijmm.2024.5364 PMC10914311 38426579

[CR8] Yu Y, Wang Z, Zheng Q, Li J (2021) FAM72 serves as a biomarker of poor prognosis in human lung adenocarcinoma. Aging 13(6):8155–8176 10.18632/aging.202625 PMC8034972 33686947

[CR7] Yu Y, Wang Z, Zheng Q, Li J (2021) GREB1L overexpression correlates with prognosis and immune cell infiltration in lung adenocarcinoma. Sci Rep 11(1):13281 10.1038/s41598-021-92695-x PMC8225624 34168239

[CR37] Yu L, Wei J, Liu P (2022) Attacking the PI3K/Akt/mTOR signaling pathway for targeted therapeutic treatment in human cancer. Semin Cancer Biol 85:69–94 10.1016/j.semcancer.2021.06.019 34175443

[CR15] Zacapala-Gómez AE, Mendoza-Catalán MA, Antonio-Véjar V, Jiménez-Wences H, Ortíz-Ortíz J, Ávila-López PA et al (2024) TET enzymes and 5hmC epigenetic mark: new key players in carcinogenesis and progression in gynecological cancers. Eur Rev Med Pharmacol Sci 28(3):1123–1134 10.26355/eurrev_202402_35349 38375718

[CR22] Zeng Y, Shi Y, Xu L, Zeng Y, Cui X, Wang Y et al (2021) Prognostic value and related regulatory networks of MRPL15 in non-small-cell lung cancer. Front Oncol 11:656172 10.3389/fonc.2021.656172 PMC8138120 34026630

[CR38] Zhang Y, Kwok-Shing Ng P, Kucherlapati M, Chen F, Liu Y, Tsang YH et al (2017) A pan-cancer proteogenomic atlas of PI3K/AKT/mTOR pathway alterations. Cancer Cell 31(6):820–32e3 10.1016/j.ccell.2017.04.013 PMC5502825 28528867

[CR14] Zhang X, Zhang Y, Wang C, Wang X (2023) TET (Ten-eleven translocation) family proteins: structure, biological functions and applications. Signal Transduct Target Ther 8(1):297 10.1038/s41392-023-01537-x PMC10415333 37563110

[CR4] Zhao Y, Yu L, Wang L, Wu Y, Chen H, Wang Q et al (2022) Current status of and progress in the treatment of malignant pleural effusion of lung cancer. Front Oncol 12:961440 10.3389/fonc.2022.961440 PMC9933866 36818672

[CR27] Zhou W, Ouyang J, Li J, Liu F, An T, Cheng L et al (2021) MRPS17 promotes invasion and metastasis through PI3K/AKT signal pathway and could be potential prognostic marker for gastric cancer. J Cancer 12(16):4849–4861 10.7150/jca.55719 PMC8247386 34234855

