# Supplementary material for: TET1-mediated DNA demethylation and transcription activation of MRPS17 induces lung adenocarcinoma through PI3K-AKT-mTOR pathway
Source: J Mol Histol. 2026 Aug 29;57(5):283. doi: 10.1007/s10735-026-10862-8 (PMC13525032; doi:10.1007/s10735-026-10862-8)
Supplement: Supplementary file 1 [file 10735_2026_10862_MOESM1_ESM.docx]

Figure S1. MRPS17 is upregulated in LUAD tissues. (A) Relative MRPS17 mRNA expression in normal lung tissues and LUAD tissues. (B) Representative western blot images showing MRPS17 protein expression in paired adjacent normal (N) and tumor (T) tissues. (C) Representative immunohistochemical staining of MRPS17 in normal lung tissue and LUAD tissue. Scale bar, 50 μm. Data are presented as mean ± SD. ***P < 0.001.

Figure S2. CpG island analysis was performed using the UCSC Genome Browser (GRCh38/hg38 assembly, Regulation group, CpG Islands track, cpgIslandExt table).

Figure S3. TET1 directly binds to the MRPS17 promoter. Data are presented as mean ± SD from three independent experiments. Statistical significance was determined using one-way ANOVA as appropriate. *P < 0.05, **P < 0.01, ***P < 0.001.

Figure S4. MRPS17 knockdown attenuated the pro-tumorigenic effects induced by TET1 overexpression. (A) qRT-PCR analysis of TET1 mRNA expression in cells transfected with the TET1 overexpression plasmid (OE-TET1) or co-transfected with OE-TET1 and si-MRPS17. (B) qRT-PCR analysis of MRPS17 mRNA expression in the indicated groups. (C) CCK-8 assay was performed to evaluate cell viability in the indicated groups at different time points. Data are presented as mean ± SD from three independent experiments. Statistical significance was determined using one-way ANOVA or two-way ANOVA as appropriate. *P < 0.05, **P < 0.01, ***P < 0.001.

Figure S5. PI3K activity assay demonstrates that MRPS17 activates the PI3K pathway in LUAD cells. Data are presented as mean ± SD from three independent experiments. Statistical significance was determined using one-way ANOVA as appropriate. *P < 0.05, **P < 0.01, ***P < 0.001.
