# Supplementary material for: TET1-mediated DNA demethylation and transcription activation of MRPS17 induces lung adenocarcinoma through PI3K-AKT-mTOR pathway
Source: J Mol Histol. 2026 Aug 29;57(5):283. doi: 10.1007/s10735-026-10862-8 (PMC13525032; doi:10.1007/s10735-026-10862-8)
Supplement: Supplementary file 2 [file 10735_2026_10862_MOESM2_ESM.docx]

Fig.2B

MPRS17	GAPDH

Fig.2H

MPRS17	GAPDH

Fig.3B

MPRS17	GAPDH

Fig.4B

MPRS17	GAPDH

Fig.6C

MPRS17	GAPDH

Fig.6E

MPRS17	TET1	GAPDH

Fig.6G

MPRS17	TET1	GAPDH

Fig.6I

MPRS17	TET1	GAPDH

Fig.7C

MPRS17	TET1	GAPDH

Fig.8C

P-AKT	P-PI3K	P-mTOR	PI3K-AKT mTOR

Fig.S1

MPRS17	GAPDH
